# Minimizing Energy Demand in the Conversion of Levulinic Acid to γ‑Valerolactone via Photothermal Catalysis Using Raney Ni

**DOI:** 10.1002/advs.202416153

**Published:** 2025-04-17

**Authors:** Roger Bujaldón, Arnau Fons, Jaume Garcia‐Amorós, Cristina Vaca, Josep Nogués, Maria José Esplandiu, Elvira Gómez, Borja Sepúlveda, Albert Serrà

**Affiliations:** ^1^ Grup d'Electrodeposició de Capes Primes i Nanoestructures (GE‐CPN) Departament de Ciència de Materials i Química Física Universitat de Barcelona Martí i Franquès, 1 Barcelona Catalonia E‐08028 Spain; ^2^ Institute of Nanoscience and Nanotechnology (IN2UB) Universitat de Barcelona Barcelona Catalonia Spain; ^3^ Instituto de Microelectrónica de Barcelona (IMB‐CNM, CSIC) Campus UAB, Bellaterra Barcelona 08193 Spain; ^4^ Grup de Materials Orgànics Departament de Química Inorgànica i Orgànica Secció de Química Orgànica Universitat de Barcelona Martí i Franquès 1 Barcelona Catalonia E‐08028 Spain; ^5^ Catalan Institute of Nanoscience and Nanotechnology (ICN2), CSIC and BIST Campus UAB, Bellaterra Barcelona E‐08193 Spain; ^6^ ICREA Pg. Lluís Companys 23 Barcelona 08010 Spain

**Keywords:** γ‐valerolactone, biomass valorization, lignocellulosic biomass, photothermo‐catalysis, Raney Ni

## Abstract

The valorization of lignocellulosic wastes emerges as a prime strategy to mitigate the global carbon footprint. Among the multiple biomass derivatives, γ‐valerolactone is particularly attractive as precursor of high‐value chemicals, biofuel, green solvent or perfumery. γ‐Valerolactone can be synthesized through a hydrogenation reaction from levulinic acid, obtained from cellulose. However, the high energy requirements of this synthetic pathway have hindered its industrial viability. To drastically reduce the reaction energy requirements, here a novel synthetic strategy, based on solvothermal‐photothermal processes using cost‐effective Raney‐Ni as photothermal catalyst, is proposed. First, the use of hydrogen gas is avoided by selecting isopropanol as a safer and greener H‐source. Second, a photothermocatalytic process is used to minimize the reaction temperature and time with respect to conventional reactions. This approach exploits the broadband optical absorption of the Raney®‐Ni, due to its highly damped plasmonic behavior, to achieve fast and efficient catalyst heating inside the reactor. The photothermal reaction required less than 2 h and just 132 °C to reach over 95% conversion, thereby drastically reducing the reaction time and energy consumption compared to conventional reactions. Importantly, these conditions granted high catalyst reusability. This solvothermal‐photothermal approach could offer a sustainable alternative for the industrial production of γ‐valerolactone.

## Graphical abstract

Upgrading lignocellulosic wastes into value‐added compounds represents an excellent option to reduce the current overreliance on fossil fuels. γ‐Valerolactone is especially attractive for its multiple applications such as biofuel or green solvent. Herein its production from levulinic acid, a biomass derivative, is proposed through a photothermocatalytic process using Raney Ni as a commercial platform and isopropanol as an alternative H‐source.

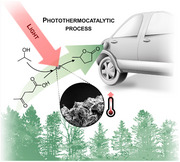

## 1 Introduction

Reducing the overreliance on fossil fuels and curbing the steady rise of CO_2_ emissions has grown into a pressing global priority to address the climate crisis. In this pursuit, biomass valorization emerges as an engaging approach for transitioning away from fossil fuels towards a more sustainable economy. Biomass originates as a residue from activities as diverse as agriculture, livestock‐related industries, and the management of forested areas, thus being the only renewable raw material that can be readily converted into organic molecules. Lignocellulosic wastes indeed hold significant interest in their derivatization to biofuels and related value‐added compounds.^[^ ^1^, ^2^, ^3^, ^4^ ^]^

The lignocellulosic biomass majorly comprises cellulose (35%−50%), hemicellulose (20%−40%) and lignin (10%–25%).^[^ ^1^, ^5^, ^6^ ^]^ The former can be transformed into hexoses or pentoses, which can be hydrolyzed into different synthetic building blocks, such as 5‐hydroxymethylfurfural (5‐HMF) and levulinic acid (LA).^[^ ^7^, ^8^, ^9^, ^10^, ^11^ ^]^ Particularly, levulinic acid represents a versatile platform towards multiple biomass derivatives of interest, such as γ‐valerolactone (GVL), which is highly promising for applications as biofuel, alternative greener solvent, precursor of biopolymers, and in perfumery.^[^ ^12^, ^13^, ^14^, ^15^ ^]^ Accessing an economically viable process for a sustainable production of γ‐valerolactone from biomass has certainly become a pivotal area of research. The synthesis of γ‐valerolactone implies the hydrogenation of the ketone group of the levulinic acid, which is generally conducted with molecular hydrogen, and its subsequent intramolecular esterification.^[^ ^4^, ^16^, ^17^, ^18^, ^19^, ^20^ ^]^ However, the production of hydrogen still mainly relies on non‐renewable sources, which critically hampers the sustainability expected from this procedure. This method also involves working under high pressure to promote the reaction, thus increasing the safety risks and complicating the requirements of the facilities and industrial plants. A well‐known strategy to circumvent these hurdles is the incorporation of alternative biomass‐derived reducing agents in which hydrogen is generated in situ. Two prime examples are formic acid, which decomposes catalytically into hydrogen and carbon dioxide, and alcohols, particularly secondary ones, which undergo a catalytic hydrogen transfer via the Meerwein−Ponndorf−Verley reaction.^[^ ^18^, ^21^, ^22^, ^23^, ^24^, ^25^, ^26^ ^]^ Formic acid is attractive since it is produced equimolarly with levulinic acid during the acid‐mediated rehydration and subsequent oxidation of 5‐HMF. Regarding alcohols as hydrogen source, even though there are a few reports employing methanol or ethanol, isopropanol outshines in terms of versatility and performance.^[^ ^18^, ^21^, ^22^, ^23^, ^24^ ^]^ Moreover, isopropanol is increasingly recognized as a green choice, due to the advances towards an industrial‐scale production with a negative carbon footprint based on the fermentation of waste gases from factories or biomass‐derived sugars.^[^ ^27^, ^28^, ^29^, ^30^, ^31^, ^32^ ^]^

Another key challenge in the γ‐valerolactone synthesis is the high energy consumption associated to the elevated temperatures required to carry out the hydrogenation reaction, which depend on the selected catalyst. Diverse noble and non‐noble metals catalysts have been reported so far,^[^ ^16^, ^17^, ^33^ ^]^ generally working at temperatures in the 200–250 °C range, making the synthesis highly energy demanding. Therefore, developing new technologies to reduce energy consumption is a major challenge. In this context, photothermal catalytic reactions could provide an interesting alternative to reduce the necessary input energy. In photothermal catalysis, the catalyst absorbs the incident light and then converts the photon energy into thermal energy by exciting vibrational states to drive the chemical reaction.^[^ ^34^, ^35^, ^36^ ^]^ Various forms of nanocarbon materials such as graphene, graphene oxide, carbon nanotubes or reduced graphene oxide have shown broadband light absorption, high stability and good thermal conductivity, although they suffer from high cost and limited activity in hydrogenation reactions.^[^ ^37^, ^38^, ^39^, ^40^, ^41^, ^42^ ^]^ As alternative, noble metal plasmonic nanoparticles can combine high catalytic activity and efficient photothermal heating endowed by their localized surface plasmon resonances.^[^ ^43^, ^44^ ^]^ However, their high cost and narrow absorption spectral bands, which reduce the photothermal efficiency in broadband light sources such as sunlight, can limit their industrial application. Therefore, it is necessary to find new cost‐effective, abundant and highly stable photothermocatalytic processes to drastically reduce the economic and environmental cost of hydrogenation reactions and, in particular, of the synthesis of γ‐valerolactone.

To reduce the energy demand of this process, herein we propose a more sustainable photothermal catalytic strategy using Raney Ni as a highly accessible and inexpensive catalyst widely recognized in hydrogenation processes, by exploiting its highly damped plasmonic behavior to generate efficient photothermal heating, even in micron size particles. We first analyze the reaction requirements under hydrothermal conditions using formic acid and isopropanol as H‐sources, showing significantly higher conversion efficiencies in the case of isopropanol, which are further improved under solvothermal conditions, reaching a 90% conversion efficiency at 160 °C after 3 h of reaction. Next, we demonstrate the drastic reduction of the energy requirements of the photothermocatalytic reaction using a near‐infrared laser diode as light source to efficiently heat the Raney Ni inside the reactor with minimal thermal losses.^[^ ^45^ ^]^ We demonstrate nearly complete photothermal conversion (>95%) of levulinic acid to γ‐valerolactone at a temperature of 132 °C after less than 2 h of reaction. In addition, owing to the mild reaction conditions, we show the high stability of Raney Ni, enabling several reaction cycles with the same catalyst without losing any catalytic efficiency. These results benchmark this photothermocatalytic process based on Raney Ni as an economically and environmentally viable solution to valorize biomass waste.

## 2 Results and Discussion

### 2.1 Characterization of the Raney Nickel Catalyst

The commercial Raney nickel microparticles were characterized as received to get further insight into their properties. The XRD pattern of the catalyst (Figure S1a, Supporting Information) revealed major diffraction peaks ascribed to the space group *Fm‐*3*m*  of the cubic system of metallic nickel [2θ = 44.5° (111), 51.8° (200), 76.4° (220)].^[^ ^46^ ^]^ Also, minor peaks could be assigned to the same space group of NiO [2θ = 37.3° (111), 43.3° (200), 63.4° (220), 75.4° (311)], 79.4° (222)], indicating a partial oxidation of the catalyst upon storage (Figure S1a, Supporting Information). Alternative phases such as NiAl alloy or Al_2_O_3_ could not be observed with this technique due to the low percentage of aluminum. XPS revealed a NiO‐enriched composition on the material surface, while also detecting the presence of Al_2_O_3_ and Ni(OH)_2_ (Figure S1b, Supporting Information).^[^ ^47^, ^48^ ^]^ FE‐SEM images displayed that the as‐received catalyst was composed of irregular and elongated microparticles presenting cracked and slightly rough surface morphology (Figure S2a, Supporting Information), as expected for Raney‐type catalysts. The specific surface area of the material obtained by BET was 21.4 m^2^ g^−1^, with an average pore width of ≈21 Å (Figure S3, Supporting Information).

### 2.2 Comparison Between H‐Sources

**Figure 1:**
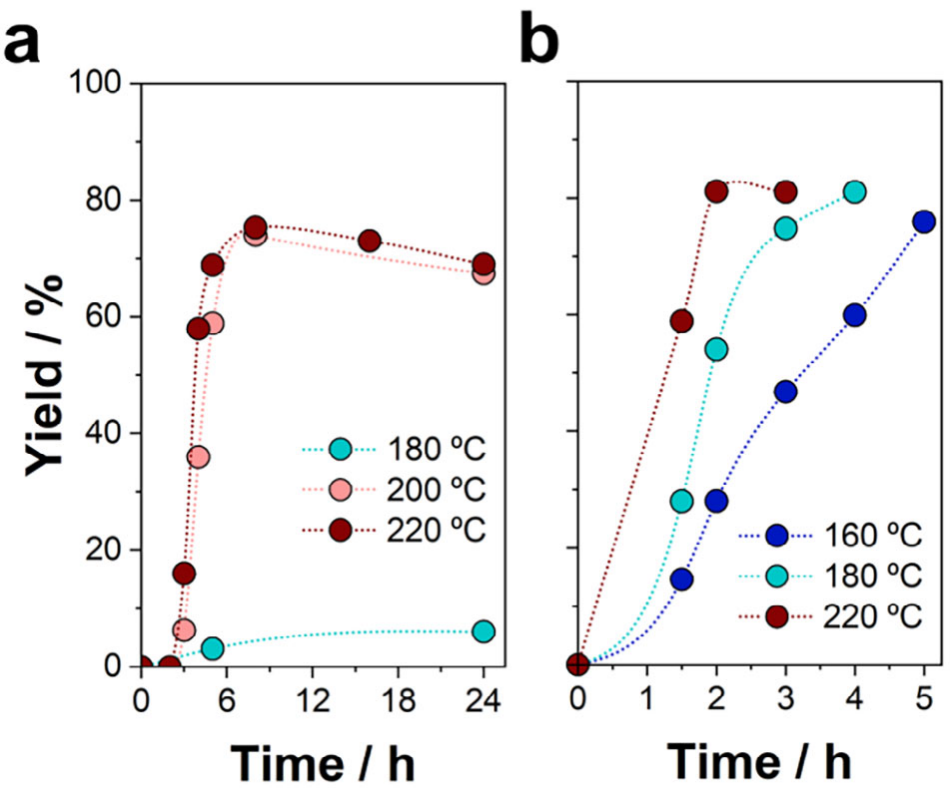
Evolution of the yield of γ‐valerolactone over time at different temperatures under hydrothermal conditions, using a) formic acid and b) isopropanol as the H‐source. Reaction conditions: catalyst mass = 120 mg; volume = 4 mL; [LA]_0_ = 0.5 M. Each condition was performed in triplicate.

As a preliminary step, we optimized the synthesis conditions with two hydrogenating agents, i.e., formic acid and isopropanol, under hydrothermal conditions employing Raney nickel as catalyst. The comparison was performed by evaluating the yield of γ‐valerolactone as a function of temperature and reaction time. The initial concentration of levulinic acid was set at 0.5 M, while maintaining an excess of either formic acid or isopropanol (2 M). **Figure** **1** shows the yield of γ‐valerolactone over time at different temperatures. As can be observed, the reaction with formic acid required substantially higher temperatures to proceed than those of isopropanol, implying an optimal performance between 200–220 °C (Figure 1a), which is in accordance with the temperatures described in the literature.^[^ ^49^ ^]^ At 200–220 °C, the reaction with formic acid took 8 hours to achieve a maximum conversion and yield of 93% and 75%, respectively. Thereafter, the yield decreases due to the raise of by‐products like valeric acid, which come from further hydrogenation processes on γ‐valerolactone.^[^ ^17^, ^50^ ^]^ In contrast, the reaction conducted at 180 °C resulted in a yield below 10% even after 24 h.

On the other hand, the use of isopropanol as the H‐source offered a significant reduction in the reaction time at 220 °C, obtaining 93% conversion and 81% yield after just 2 h of reaction (Figure 1b). Unlike formic acid, isopropanol permitted a substantial decrease in the operating temperature, attaining yields ≈80% after 3 h at 180 °C, or after 5 h at 160 °C. These findings agree with the promising results described for the conversion of alkyl levulinates with isopropanol under mild conditions.^[^ ^51^, ^52^ ^]^ Therefore, isopropanol as a hydrogen source promotes a faster and more sustainable procedure, arising as the optimal choice for this synthetic strategy.

### 2.3 Solvothermal Reaction

The obtained results for isopropanol as the H‐source pointed towards its use as solvent to achieve a more efficient conversion under even milder conditions. To demonstrate the effect of the isopropanol, we compared the results under hydrothermal conditions with the yield of γ‐valerolactone using isopropanol as solvent at 160 °C (Figure S4, Supporting Information). Indeed, the yield almost doubles with this step, going from 47% to 91% after 3 h. Therefore, we further optimized the solvothermal conditions as a function of temperature, time, catalyst mass and levulinic acid concentration.

**Figure 2:**
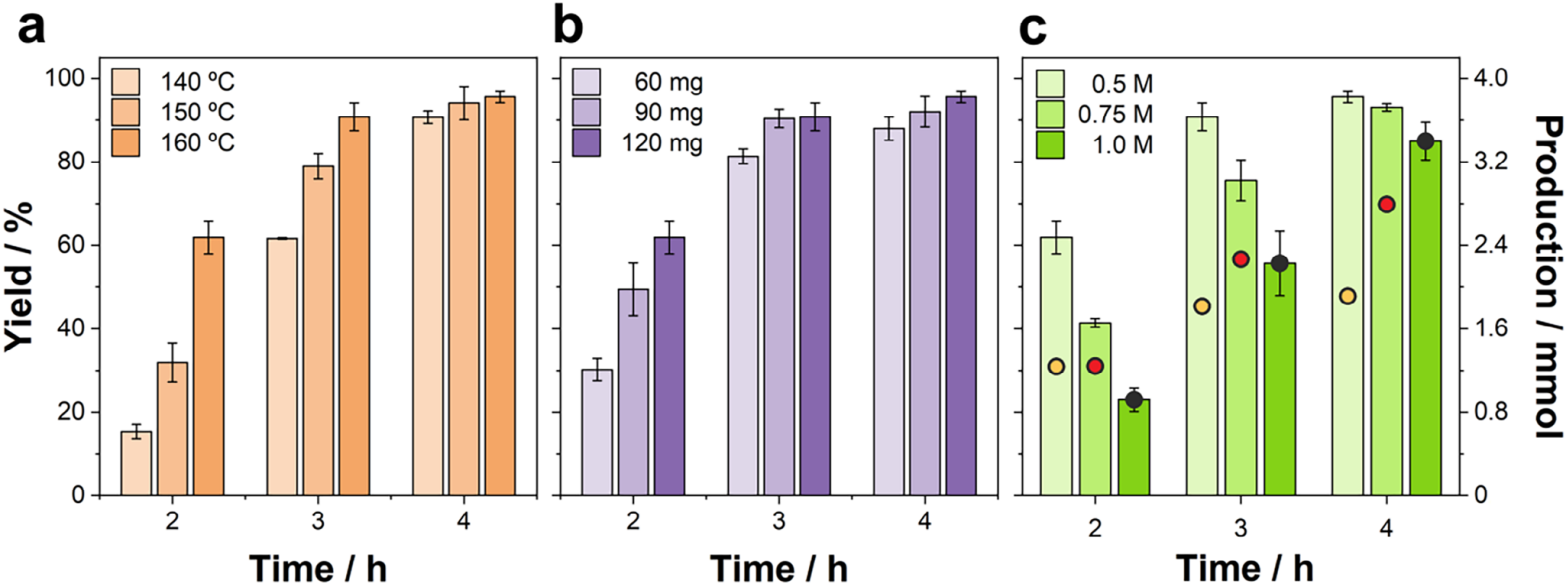
Evaluation of the yield of γ‐valerolactone obtained under solvothermal conditions in isopropanol in an autoclave, in the dark, at different reaction times, depending on: a) the oven temperature, b) the amount of catalyst and c) the initial concentration of levulinic acid (the total production in mmol is indicated with circles and associated with the right axis). Unless otherwise stated, the reference reaction conditions imply: catalyst = 120 mg; [LA]_0_ = 0.5 M; T = 160 °C. Each condition was performed in triplicate.

As observed in **Figure** **2**, the yield raised from 60% to 96% when increasing the reaction time from 2 to 4 h at 160 °C under solvothermal conditions. Temperature remained a key factor from a kinetic point of view, as reactions were considerably slower for temperatures below 160 °C (Figure 2a). This was especially noticeable after 2 h of reaction at 140 and 160 °C, which resulted in yields of 15% and 62%, respectively. Nevertheless, the reaction could be nearly completed at 140 °C after 4 h, with a yield of 91%. Therefore, employing isopropanol as solvent enables an additional 20 °C reduction with respect to hydrothermal conditions. Second, the relationship between yield and catalyst ratio was further explored (Figure 2b). The decrease in catalyst quantity led to a reduction in reaction rate, but the reaction was nearly completed in 3 h at 160 °C, when the amount of catalyst was equal or higher than 90 mg. Lastly, Figure 2c represents the yield and production in mmol of γ‐valerolactone with respect to different levulinic acid concentrations. As observed, after 2 h of reaction, the γ‐valerolactone production was almost similar regardless of the levulinic acid concentration, producing 1.2 mmol with 120 mg of catalyst. The increase in the reaction time for a concentration of levulinic acid of 1 M enabled rising the amount of γ‐valerolactone, obtaining ≈2.3 mmol after 3 h of reaction, and 3.4 mmol after 4 h, with a yield of 85%. While higher levulinic acid concentrations led to equally successful reactions, they required a substantial increase in reaction time and catalyst mass to attain nearly complete conversions (Figure S5, Supporting Information). Therefore, commercially available Raney Ni catalyst under solvothermal conditions offers milder and more scalable reactions than conventional reactions.

### 2.4 Photo‐Thermo‐Catalytic Reaction

Despite the considerable improvement attained in the conversion from levulinic acid to γ‐valerolactone under solvothermal conditions using isopropanol as hydrogen source and solvent, a photo‐thermo‐catalytic strategy can offer a remarkable additional reduction of the energy requirements, both in terms of reaction temperature and time. Here we demonstrate that Raney Ni can be exploited as an efficient photothermal heating catalyst for the transformation of levulinic acid into γ‐valerolactone, thus acting as the local heat source by efficiently absorbing the incident photons. The photothermal mechanism in the Raney Ni micropowder is given by its highly damped plasmonic behavior. Even though the surface of Raney Ni is chemically complex, showing both oxides and metallic components, the visible and NIR light is mainly absorbed by the metallic Ni and the Ni/Al alloy. These materials are characterized by a negative real part of the permittivity in the visible and near infrared (typical of plasmonic metals), but a much higher imaginary part of the permittivity than typical plasmonic materials (e.g., Ag and Au), which provides the highly damped plasmonic behavior. As a result, light can penetrate deeper into highly damped metals, thereby simultaneously enhancing the light absorption and minimizing the scattering.^[^ ^31^, ^46^, ^47^ ^]^ These features are very relevant in the case of the Raney Ni micropowder due to the large grain size (10–50 µm range), which prevents the excitation of localized surface plasmon resonances in the visible and NIR ranges. However, the highly damped plasmonic behavior and associated enhanced light penetration into the metal enables Raney Ni showing a broadband and intense absorption and reduced scattering in both the visible and NIR spectral ranges. Actually, this behavior can be visually perceived by the nearly black appearance of the Raney Ni powder in liquid.

Due to the large size of the Raney Ni microparticles and their fast sedimentation, which prevents standard absorbance measurements, we directly analyzed the photothermal heating efficiency (which is proportional to the optical absorption of the particles). Moreover, due to the difficulty to determine the actual temperature of the microparticles in solution, the photothermal characterization was performed in 100 mg of dry Raney Ni powder homogeneously distributed in an area of 0.64 cm^2^ (Figure S5, Supporting Information). In the photothermal experiments we illuminated the dry powder with 3 different light sources: a white LED with broadband spectrum (400–600 nm), and two different lasers with emission at 808 and 1064 nm. Due to the high reactivity of the Raney Ni powder in air, we employed low light intensities during the photothermal characterization (100 mW cm^−2^). The light sources illuminated the sample in normal incidence and the photothermal temperature increase was monitored with a thermal camera positioned in an angle of ≈20 degrees (Figure S6, Supporting Information). In these measurements we considered the infrared emissivity of the Raney Ni powder, which was previously determined, obtaining a value of 0.83. As can be seen in Figure S6 (Supporting Information), the Raney Ni exhibited efficient photothermal heating, but showing increasing efficiency toward the near‐infrared, probably due to the large size of the microparticles and the higher scattering at shorter wavelengths.

Considering this result, a near infrared laser diode with emission at 915 nm was selected to assess the photothermal catalytic response of Raney Ni for the synthesis of γ‐valerolactone. The photothermal catalytic efficiency was analyzed by illuminating the bottom of a glass Tinyclave reactor with the collimated 915 nm laser beam (diameter ≈2 cm). It is worth noticing that the infrared thermometer, pointing at the lateral surface of the glass reactor, estimates the average temperature of the liquid solution. Since the Raney Ni micropowder acts as the heat source inside the reactor, its temperature must be higher than the temperature detected by the thermometer. However, currently there is no technically simple method to determine the actual temperature of the Raney Ni microparticles inside the sealed reactor under photothermal heating, which induces intense convection movement of the particles. Moreover, the temperature of the liquid solution is much more relevant from the thermodynamics point of view, due to the much higher mass and heat capacity of the reactants compared to the metallic catalyst. Consequently, the majority of the supplied energy (either internally by photothermal effect or externally by the oven) is used to increase the temperature of the solution. It is also important to point out that the heat capacity of the liquids reactants also substantially increase with the temperature, whereas it is approximately equal for the metals in the range of temperatures in which the reaction takes place. Therefore, in this case, the average liquid temperature provides more relevant information of the energy consumption during the reaction process.

The programmed temperature set point was maintained by automatically adjusting the laser power. The optimal initial laser power for a reactant volume of 4 mL was between 15 and 18 W, as lower values were insufficient to reach the required temperature set point in a practical extent of time (Figure S7a, Supporting Information), whereas higher intensities could damage the catalyst surface and limit their long‐term reusability, as observed by SEM (Figure S2b,c, Supporting Information). On the other hand, the heating curves only slightly depended on the amount of catalyst, which reflects that the majority of the incident light was already absorbed by the first layer of Raney Ni microparticles, even by the lowest amount of catalyst (60 mg) (Figure S7b, Supporting Information).

Interestingly, when levulinic acid was added to the reactor, the heating curves were significantly different than those of only isopropanol (Figure S8a, Supporting Information). Specifically, with 120 mg of catalyst, pure isopropanol took ≈20 min to attain 140 °C, while a solution with levulinic acid (0.5 M) reacting in situ needed 80 min. This is due to the absorbed heat by the overall endothermal process (Δ_*process*_ *H*  =  690± 4 kJ kg^−1^).^[^ ^48^ ^]^ In contrast, the photothermal heating without the Raney Ni microparticles is much lower, thereby demonstrating that the photothermal catalytic effects can be ascribed to the Raney Ni.

**Figure 3:**
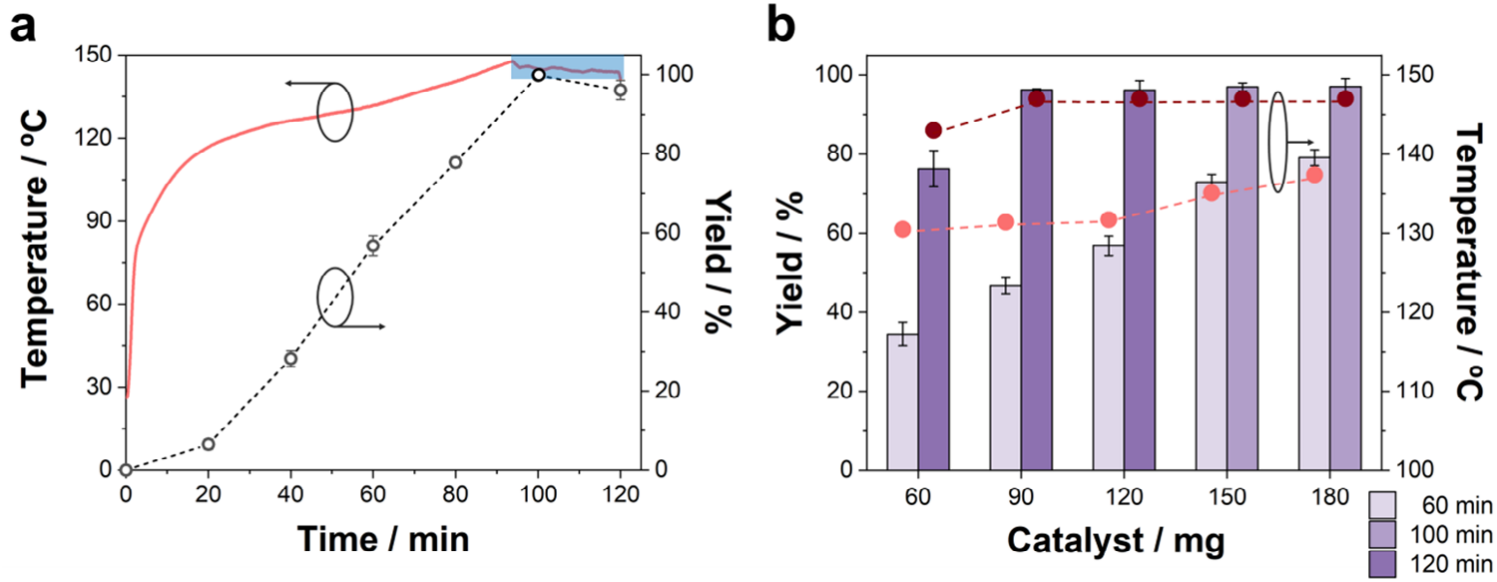
Photothermal catalytic experiments. a) Evolution of the temperature and yield of γ‐valerolactone as a function of reaction time using 120 mg of catalyst. b) Yield of the produced γ‐valerolactone (bars) and maximum obtained temperature [dots; light symbols – 60 min, and dark symbols – longer reaction times (100 or 120 min)] depending on the amount of catalyst for two different reaction times. Note that the longer reactions were conducted for 120 min for 60–120 mg of catalyst, while they were carried out for 100 min in the cases of 150 and 180 mg because of the faster completion of the reaction for higher concentrations. The reactions were conducted under laser‐induced heating and solvothermal conditions in isopropanol, [LA]_0_ = 0.5 M. The laser power was maintained constant at 18 W until the reactor reached a pressure 10 bars, and then the power was reduced to keep a stable temperature and avoid overpressure (blue region in (a)). Each result was performed in triplicate.

Considering the high photothermal efficiency of Raney Ni, the reaction yield was first analyzed under a constant light power (18 W) for different reaction times (**Figure** **3**a), and it was correlated with the heating curve versus time. The laser power of 18 W induced a steady temperature rise until the reactor reached a pressure close to the maximum safety value (10 bar) of the reactor, at which the power was reduced to keep a stable temperature and to avoid overpressure (highlighted in blue in Figure 3a). The reaction yield was remarkably higher under photothermal actuation compared to the conventional autoclave reactor, as it only required 60 min to furnish a 57% γ‐valerolactone yield with a maximum temperature of 130 °C, and 100 min for full completion, with a maximum temperature of 147 °C. For longer times, the yield slightly decayed due to the emergence of different by‐products.

**Figure 4:**
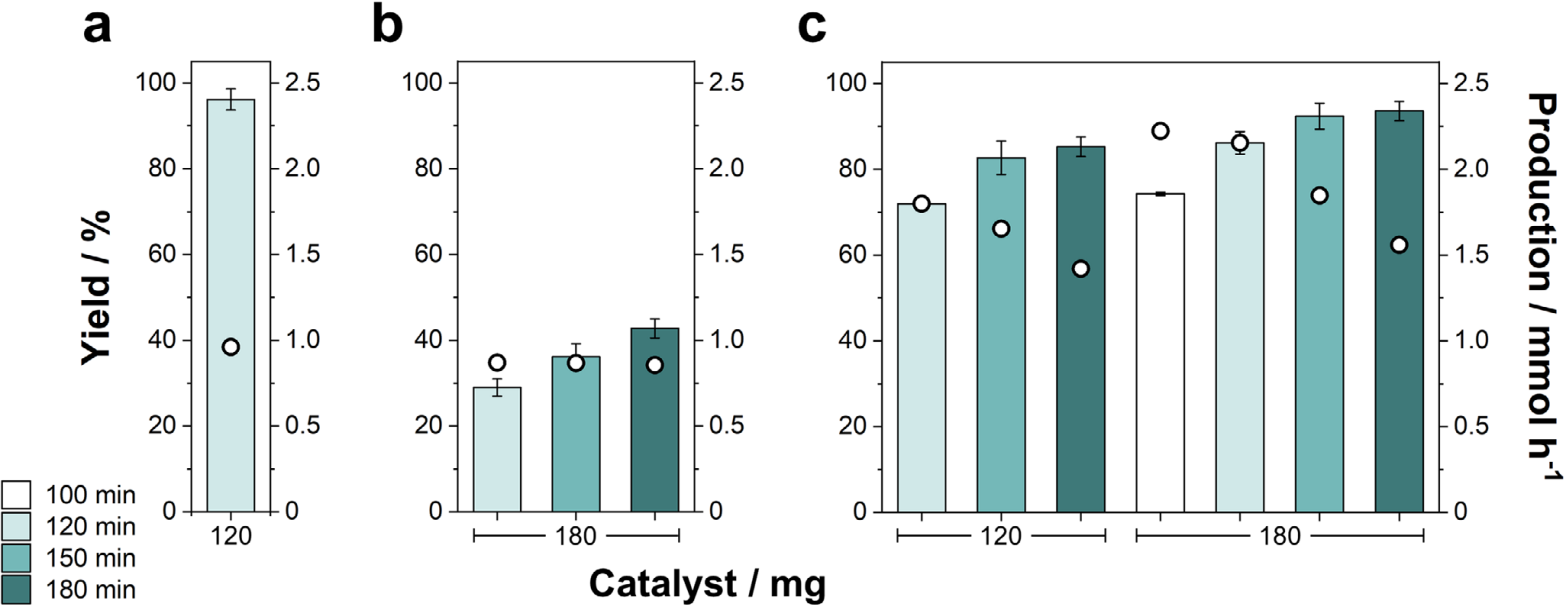
Evaluation of the yield (bars) and production per hour (dots) of γ‐valerolactone at different reaction times under photothermal conditions (T_max_ = 132 °C; laser initial power = 18 W): a) [LA]_0_ = 0.5 M; V_sol_ = 4 mL; b) increasing [LA]_0_ to 1.5 M (V_sol_ = 4 mL), and c) increasing V_sol_ to 10 mL ([LA]_0_ = 0.5 M). Each condition was performed in triplicate.

The impact of the amount of catalyst (from 60 to 180 mg) on the reaction yield and maximum temperature was also surveyed using the same illumination conditions (Figure 3b). The yields after 1 h of reaction showed a linear increase with the amount of catalyst, rising from 35% to 79% with 60 and 180 mg of catalyst, respectively. As the heating curves were nearly identical (Figure S7b, Supporting Information), the yield improvement was mainly related to the increase of available catalytic surface. Except for the reaction with the lowest quantity of catalyst (i.e., 60 mg), all reactions were accomplished within 2 h, with yields beyond 96%, and reaching similar maximum temperatures. Interestingly, for 120 mg of catalyst nearly total conversions could be achieved in only 100 min. Hence, the photothermal strategy grants a remarkable reduction of the reaction time thanks to the role of Raney Ni as efficient heat source inside the reactor, but it also suggests that the reaction can admit even lower working temperatures. To demonstrate this, the reaction was carried out during 2 h at a maximum temperature of 130 °C, which was maintained by automatically adjusting the laser power (Figure S8b, Supporting Information), resulting in an excellent 93% yield. This value could be further increased to 97% by rising the set point to just 132 °C (**Figure** **4**a). These results demonstrate the superior energy efficiency of the photothermal catalytic strategy, both in terms of temperature and reaction time.

The scalability of the photothermal reaction was studied by increasing either the concentration of levulinic acid or the volume of solution at a fixed concentration of levulinic acid (0.5 M). As shown in Figure 4b,c, escalating the volume from 4 to 10 mL yields a more efficient process than increasing the LA concentration, leading to a γ‐valerolactone molar production (2.2 mmol h^−1^). Notably, increasing the volume of the solution from 4 to 10 mL did not have any detrimental effect on the yield (over 80% for reaction times longer than 120 min), but it increased considerably the γ‐valerolactone molar production rate form 0.9 mmol h^−1^ (4 mL) to 2.2 mmol h^−1^ (10 mL), i.e., an outstanding 2.5‐fold enhancement.

These results indicate that Raney Ni micropowder acts as a photothermal heating catalyst to achieve the faster conversion of levulinic acid into γ‐valerolactone at lower temperatures, due to the following reasons. First, the reaction also takes place under pure thermocatalytic conditions in the conventional oven, although at longer times and higher overall solution temperature. Second, the faster and milder overall photothermal reaction conditions are due to the highly damped plasmonic behavior of Raney Ni, which enables an efficient local photothermal heating of the reactants surrounding the microparticles, thereby activating the catalytic reaction. As the Raney Ni powder acts as the local heat source, its local temperature is higher than the bulk liquid (isopropanol + levulinic acid), the reaction can be generated at a lower global temperature inside the reactor. As a result of the faster photothermal heating inside the reactor and the higher temperature of the Raney Ni particles, the complete chemical conversion takes shorter times compared to the conventional heating in the oven. Third, the only semiconducting materials in the Raney Ni micropowder are Ni oxides present at the surface, whose bandgap is large (3.5–4 eV), whereas a low energy NIR source laser for heating (915 nm, i.e., 1.35 eV) has been employed. Consequently, the activation of photocatalytic effects is not expected under these conditions.

Regarding the reaction mechanism, it should be mentioned that the conversion of isopropanol to acetone during the reaction is strongly related to the production of γ‐valerolactone. This seemingly confirms that the hydrogenation transfer with isopropanol proceeds through the Meerwein−Ponndorf−Verley mechanism, which is illustrated in Scheme S1 (Supporting Information).^[^ ^57^ ^]^

A comparative analysis of catalytic performance was conducted to contextualize our results within the existing literature. **Table** **1** summarizes representative studies on the hydrogenation of levulinic acid to γ‐valerolactone using isopropanol as the hydrogen source, demonstrating the competitive efficiency of our catalyst in terms of reaction time or temperature conversion, yield, and turnover frequency.

**Table 1:** Comparison of various catalysts for the production of γ‐valerolactone from levulinic acid using isopropanol as H‐source.

Catalyst	Cat. Mass / mg	[LA] / M	Cat. dose ^a^ ^)^	Time / h	T_max_ / °C	Conv. / %	Yield / %	TOF ^b^ ^)^	Refs.
Hf@SB‐MSA	100	0.09	58.1	12	180	100	99.1	1.4	[58]
Zr(OH)_4_	250	0.17	58.1	2	200	97.5	62.3	5.4	[59]
MnOOH	100	0.20	38.7	18	220	100	87.2	1.3	[60]
Pt/TiO_2_	10	0.09	58.1	15	80	100	91	1.1	[23]
Pt/TiO_2_ ^c^ ^)^	10	0.09 ^f^ ^)^	58.1	12	30	100	99	1.4	[23]
Hf‐USY	50	0.20	50	10	150	98.5	95.7	1.9	[61]
CePO_4_/zeolite	100	0.17	100	24	140	97	78	0.3	[62]
Ru‐PC	30	0.20 ^g^ ^)^	30.4	4	120	99.5	90.6	7.4	[24]
Ru/UCN	40	0.20	40	12	100	100	99.8	2.1	[63]
Pt_(5 wt.%)_/C	100	0.05	200	0.5	160	95	77	7.7	[64]
Ru_(5 wt.%)_/C	100	0.05	200	0.5	160	99	82	8.2	[64]
Ru_(5 wt.%)_/C ^d^ ^)^	100	0.05	200	0.5	160	>99	99	9.9	[64]
GluPC‐Zr	50	1.87	10	12	190	>99	98.1	8.2	[65]
HPW@MOF	50	0.20	50	6	160	>99	86	2.9	[66]
Hf‐ATMP	200	0.25	200	4	150	>99	98	1.2	[67]
Raney Ni	120	0.50	60	4	160	98	88	3.7	This work
Raney Ni ^e^ ^)^	120	0.50	60	1.7	132	>99	99.8	9.8	This work
Raney Ni ^e^ ^)^	180	0.50	36	1.5	132	97	92.4	10.3	This work

^a)^ Catalyst dose (mg_cat_ mmol_LA_^−1^);

^b)^ Turnover frequency (mmol_GVL_ h^−1^ g_cat_^−1^);

^c)^ Photocatalytic process via irradiation with a LED (365 nm);

^d)^ Microwave heating;

^e)^ Photo‐thermo‐catalytic process through laser irradiation;

^f)^ Mixture of isopropanol:water (4:1 v/v) as solvent;

^g)^ Mixture of isopropanol:water (1:1 v/v) as solvent.

**Figure 5:**
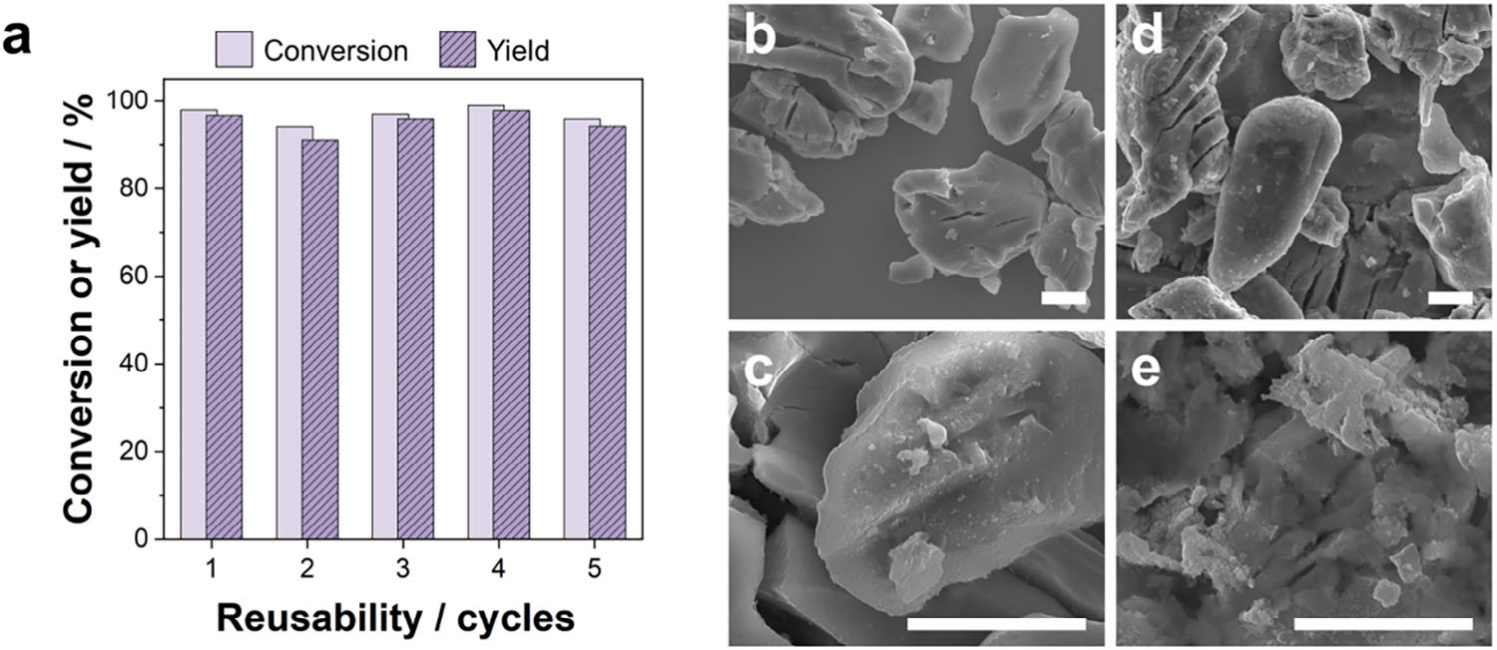
Reusability and stability analyses performed under laser‐induced heating and solvothermal conditions in isopropanol: a) conversion of levulinic acid and yield of γ‐valerolactone through 5 consecutive cycles. FE‐SEM images comparing the morphology of b,c) the pristine catalyst and d,e) the reused one (scale bar 5 µm). Conditions: catalyst = 120 mg; [LA]_0_ = 0.5 M; T_max_ = 132 °C; laser initial power = 18 W.

Finally, we studied the reusability of the catalyst, which is a key aspect to ensure the sustainability of the process. Here, the magnetic character of the catalyst was exploited for its easy recovery and separation from the reaction products. **Figure** **5**a shows that the conversion of levulinic acid and yield of γ‐valerolactone was maintained, with yields exceeding 90%, after 5 consecutive reaction cycles, thus withstanding the high potential of this synthetic strategy. The impact of successive photo‐thermo‐catalytic reactions onto the surface, morphology and structure of the catalyst was evaluated by means of FE‐SEM (Figure 5b–e), XRD and XPS (Figure S9, Supporting Information). As can be observed, the surface of the catalyst becomes rougher and cracked upon usage, potentially increasing its available surface area. Moreover, its composition and structure remained unaltered after the recyclability tests. The concentration of metal ions leaked into the reaction medium accumulated throughout 5 cycles, quantified via ICP‐OES, was in the ppm scale (Figure S9, Supporting Information), again underscoring the catalyst stability and reusability.

## 3 Conclusions

In summary, our study presents a highly sustainable procedure for the production of γ‐valerolactone, using Raney Ni as a highly accessible and inexpensive catalyst, based on: i) substituting hydrogen gas with greener alternatives (isopropanol), and ii) reducing the energy cost of the process by a photothermal actuation. Under hydrothermal conditions, isopropanol outperformed formic acid as a H‐source, enabling ≈ 80% conversion after 5 h of reaction at 160 °C. Indeed, the use of isopropanol as solvent enabled an additional improvement, obtaining ≈90% yield after 3 h under analogous conditions. Remarkably, the photothermocatalytic solvothermal approach, exploiting the highly‐damped plasmonic behavior of Raney Ni, enabled a significant reduction of the reaction temperature and time due to the efficient optical heating of the Raney Ni inside the reactor. Under a constant laser power of 18 W, this strategy provided a full conversion to γ‐valerolactone in only 100 min with a maximum temperature of 147 °C. The system was further optimized to operate under temperature‐regulated conditions (T_max_ of 132 °C) to reduce the energy consumption, achieving a remarkable 97% yield after 120 min. Importantly, the system is certainly scalable, admitting increasing reaction volumes and even providing substantially higher γ‐valerolactone production rates (up to 2.2 mmol h^−1^). Therefore, the achieved reduction of energy consumption is due to: i) the use of isopropanol as hydrogen source under solvothermal condition, which drastically decreases the reaction temperature and time with respect to the formic acid under hydrothermal condition, and ii) the photothermal heating inside the reactor, which enables further reduction of the reaction temperature and time. Even though the energy consumption of the conventional oven and the laser light energy can be quantified, the comparison would not be fair, as a much larger volume is heated in the oven compared to the photothermal reactor. Nevertheless, the reduction in reaction temperature and time in the photothermal heating reaction clearly indicates a lower energy requirement. The energy demand could be further reduced by using submicron Raney Ni particles, due to the increase in the overall surface area and the improved light absorption offered by the lower light scattering.

On the other hand, the mild conditions of this photothermal strategy ensures the reusability of the catalyst, allowing excellent γ‐valerolactone yields (over 94%) after 5 cycles, with minimal modification of the catalyst structure and negligible leakage of metals into the medium. Therefore, the proposed photothermal catalytic production of γ‐valerolactone using isopropanol as hydrogen source and solvent and cost‐effective Raney Ni circumvents the high energy cost of current γ‐valerolactone synthetic processes, which is the main limitation towards its industrialization. The energy costs could be further reduced by exploiting the broadband spectral absorption of the Raney Ni, thereby opening the path to its solar photothermal synthesis towards a large‐scale and sustainable biomass revalorization.

## 4 Experimental Section

### Synthesis and Characterization

*Reagents and chemicals*: The reagents employed include levulinic acid (Thermo Scientific, 98%), formic acid (Sigma–Aldrich, >95%) and isopropanol (Panreac, technical grade). γ‐Valerolactone (Thermo Scientific, 98%) was used for identification and calibration. Raney Ni microparticles (Sigma–Aldrich, 2800, slurry in 50 wt.% H_2_O, composition: 90% Ni, 9.5% Al, and 0.5% Fe) was selected as catalyst. All reagents were used as received.

### Characterization

The crystalline structure of the as‐received and reutilized Raney Ni microparticles was elucidated via X‐ray diffraction (XRD, D8 Advance, Bruker AXS, Germany). The chemical composition was determined by X‐ray photoelectron spectroscopy (XPS, PHI Quantera SXM), using monochromated Al Kα X‐rays with a 200 µm spot size in ultra‐high vacuum. The composition and morphology were analyzed through field‐emission scanning electron microscopy (FE‐SEM, S4800II, Hitachi, Japan) coupled with energy‐dispersive X‐ray spectroscopy (EDX) for enhanced elemental analysis. The specific surface area was determined via the Brunauer–Emmett–Teller (BET) method from N_2_ adsorption‐desorption isotherms at 77 K, using a Micrometrics Tristar‐II equipment. The release of metallic ions into the reaction medium was quantified via inductively coupled plasma optical emission spectroscopy (ICP‐OES) using an Optima 8300 Perkin Elmer equipment. The photothermal experiments were carried out with a fiber coupled laser diode with emission at 915 nm (Aerodiode 915LD‐3‐4‐2) whose output beam was collimated to a spot with a diameter of 2 cm. The collimated laser beam illuminated the bottom of a transparent borosilicate glass Tinyclave Reactor (Büchi Glass AG) with a volume of 25 mL. The reactor integrated a barometer to control the internal pressure and a safety valve, which opens if the pressure reaches 10 bars. The temperature was monitored with an IR thermometer (OPTRIS CT) pointing at the lateral side of the glass in an area of ≈1 mm^2^. The analog signal of the thermometer was acquired with a data acquisition card (NI DAQ USB‐6001) and monitored with a Labview program that also controlled the laser output power to maintain the selected temperature set point. The remaining lateral surface of the reactor was covered with a thermal insulating film (VITCAS) to minimize the heat losses. The reactor was mounted on a custom‐made temperature resistant PEEK support.

### Synthetic Procedure

The initial γ‐valerolactone synthesis experiments were conducted under hydrothermal conditions in a Teflon reactor placed into a 50 mL autoclave, utilizing 4 mL of the reaction solution (0.5 M of levulinic acid and 2 M of either formic acid or isopropanol in Milli‐Q water) with 120 mg of catalyst. The catalyst was weighted as a slurry for each experiment, and the mass values specified throughout this work refer to the weight of dry microparticles, estimated as a 1:1 microparticle:H_2_O wt/wt ratio. The second stage involved solvothermal conditions, using isopropanol as hydrogen source and solvent, and were similarly carried out with 4 mL of the reaction solution (0.5–3 M of levulinic acid in isopropanol, as specified in each case) and different amounts of catalyst. Prior to sealing the reactor, all solutions underwent 5 min purge with nitrogen. Subsequently, the autoclave was introduced into the pre‐stabilized oven set at the designated temperature. Upon completion of the programmed reaction time, the reactor was removed from the oven, allowed to cool for 20 min, and finally submerged into water to speed up the cooling process to room temperature. The resulting reaction mixture was diluted with Milli‐Q water and separated from the catalyst with the assistance of an external magnet.

In the photothermal experiments conducted under laser irradiation, the selected amount of catalyst and 4 mL of the reaction solution (0.5–1.5 M of levulinic acid in isopropanol, as specified in each case) were introduced into a glass Tinyclave reactor of 25 mL, which was purged for 5 min with nitrogen. The temperature of the reactor was monitored throughout the experiment with the infrared thermometer pointing at the lateral surface of the glass reactor. The temperature readout enabled the control of the reactor temperature and pressure by tuning the output power of a fiber coupled laser diode with emission at 915 nm. The laser light was collimated in a spot of 2 cm diameter that illuminated the bottom of the glass reactor. The laser output power was initially set at 18 W until reaching the desired T value, which was automatically maintained by fine‐tuning the laser power.

For the reusability tests, the catalyst was washed with isopropanol thrice and dried with nitrogen. The reactor was immediately filled with 4 mL of the reaction solution, purged with nitrogen for 5 min and sealed.

The reaction enthalpy was measured using a DSC 822C from Mettler Toledo with 100 µL crucibles. The temperature was increased from ambient to 132 °C at a heating rate of 2 K min^−1^. Then, the temperature has been kept at 132 °C during 2 h experiment in order to assure the entire reaction's conversion. The experiment was performed under 50 mL min^−1^ N_2_ flow.

### Sample Analysis

The composition of each sample was analyzed by liquid chromatography (HPLC) employing a Waters Alliance 2795 instrument coupled with a PDA detector (Waters 2996) and a mass detector (Waters ZQ 2000). Prior to injection into the chromatograph (1.5 µL), the solution was centrifuged at 6000 rpm for 15 min and filtered using 0.22 µm syringe filters to eliminate any solid residue. The mobile phase comprised a mixture of water and acetonitrile (starting ratio of 9:1 v/v) with 0.1% formic acid, and an ACQUITY CSH C18 column (1.7 µm, 3.0×50 mm) was selected. The concentration of the reagent and products were quantified after the equipment calibration, allowing the determination of the conversion of levulinic acid and yield of γ‐valerolactone using the following formulae:

(1) $$\mathrm{Conversion}=\left({\left[\mathrm{LA}\right]}_{0}-{\left[\mathrm{LA}\right]}_{t}\right)\times 100/{\left[\mathrm{LA}\right]}_{0}$$

(2) $$\mathrm{Yield}={\left[\mathrm{GVL}\right]}_{t}\times 100/{\left[\mathrm{LA}\right]}_{0}$$

in which [LA]_0_ and [LA]_t_ denote the initial and final concentrations of levulinic acid, respectively, and [GVL]_t_ represents the final concentration of γ‐valerolactone.

## Conflict of Interest

The authors declare no conflict of interest.

## Supporting information



Supporting Information

## Data Availability

The data that support the findings of this study are available from the corresponding author upon reasonable request.
