# Supplementary material for: Minimizing Energy Demand in the Conversion of Levulinic Acid to γ‑Valerolactone via Photothermal Catalysis Using Raney Ni
Source: Adv Sci (Weinh). 2025 Apr 17;12(21):2416153. doi: 10.1002/advs.202416153 (PMC12140316; doi:10.1002/advs.202416153)
Supplement: Supplementary file 1 — Supporting Information [file ADVS-12-2416153-s001.docx]

**Supporting information**

**Minimizing energy demand in the conversion of levulinic acid to γ‑valerolactone and its photothermal catalysis using Raney Ni**

Roger Bujaldón^1,2&^, Arnau Fons^3&^, Jaume Garcia-Amorós^2,4^, Cristina Vaca^3,5^, Josep Nogués^5,6^, Maria José Esplandiu^5^, Elvira Gómez^1,2^, Borja Sepúlveda^3,^*, Albert Serrà^1,2,^*

^1^ Grup d’Electrodeposició de Capes Primes i Nanoestructures (GE-CPN), Departament de Ciència de Materials i Química Física, Universitat de Barcelona, Martí i Franquès, 1, E-08028, Barcelona, Catalonia, Spain.

^2^ Institute of Nanoscience and Nanotechnology (IN^2^UB), Universitat de Barcelona, Barcelona, Catalonia, Spain.

^3^ Instituto de Microelectrónica de Barcelona (IMB-CNM, CSIC), Campus UAB, 08193 Bellaterra, Barcelona, Spain

^4^ Grup de Materials Orgànics, Departament de Química Inorgànica i Orgànica, Secció de Química Orgànica, Universitat de Barcelona, Martí i Franquès 1, E-08028, Barcelona, Catalonia, Spain

^5^ Catalan Institute of Nanoscience and Nanotechnology (ICN2), CSIC and BIST, Campus UAB, E-08193 Bellaterra, Barcelona, Spain

^6^ ICREA, Pg. Lluís Companys 23, 08010 Barcelona, Spain

^&^ Equal contribution

**Corresponding author:** [borja.sepulveda@csic.es](mailto:borja.sepulveda@csic.es) (B.S.), [a.serra@ub.edu](mailto:a.serra@ub.edu) (A.S.)

**
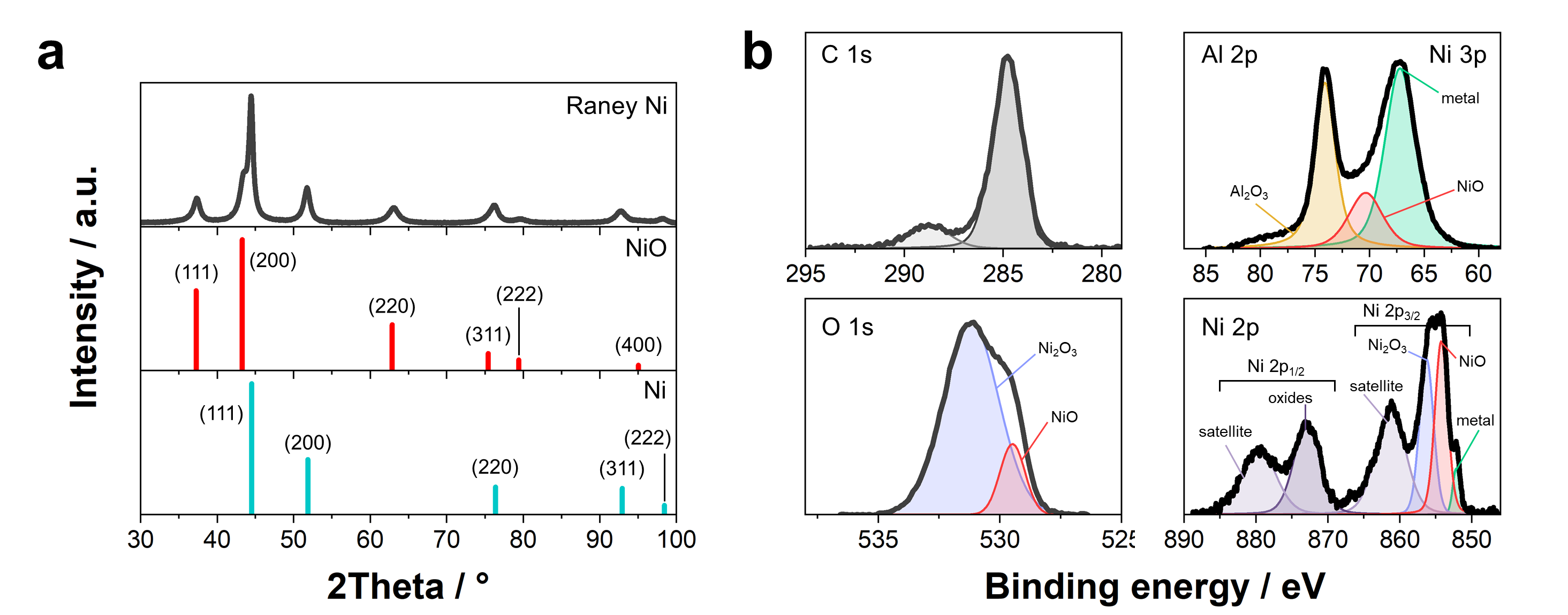
**

**Figure S1:** Characterization of the as-received Raney Ni microparticles by means of: (a) XRD, compared with metallic Ni (JCPDS card No. 04-850) and NiO (JCPDS card No. 47-1049) standards, and (b) XPS.


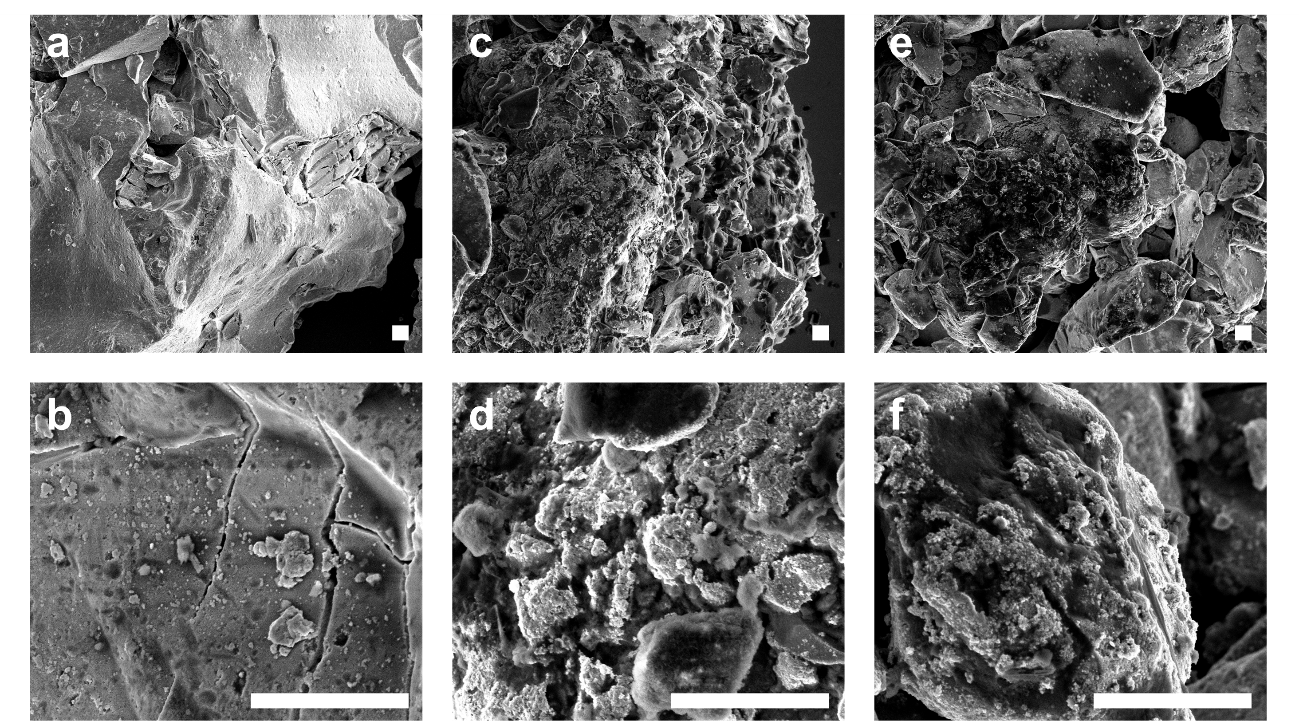


**Figure S2:** FE-SEM images displaying Raney Ni microparticles: (a,b) as received, (c,d) irradiated at a laser power of 18 W for 2 h and (e,f) irradiated at a laser power of 21.2 W for 2 h. Scale bar 5 µm.


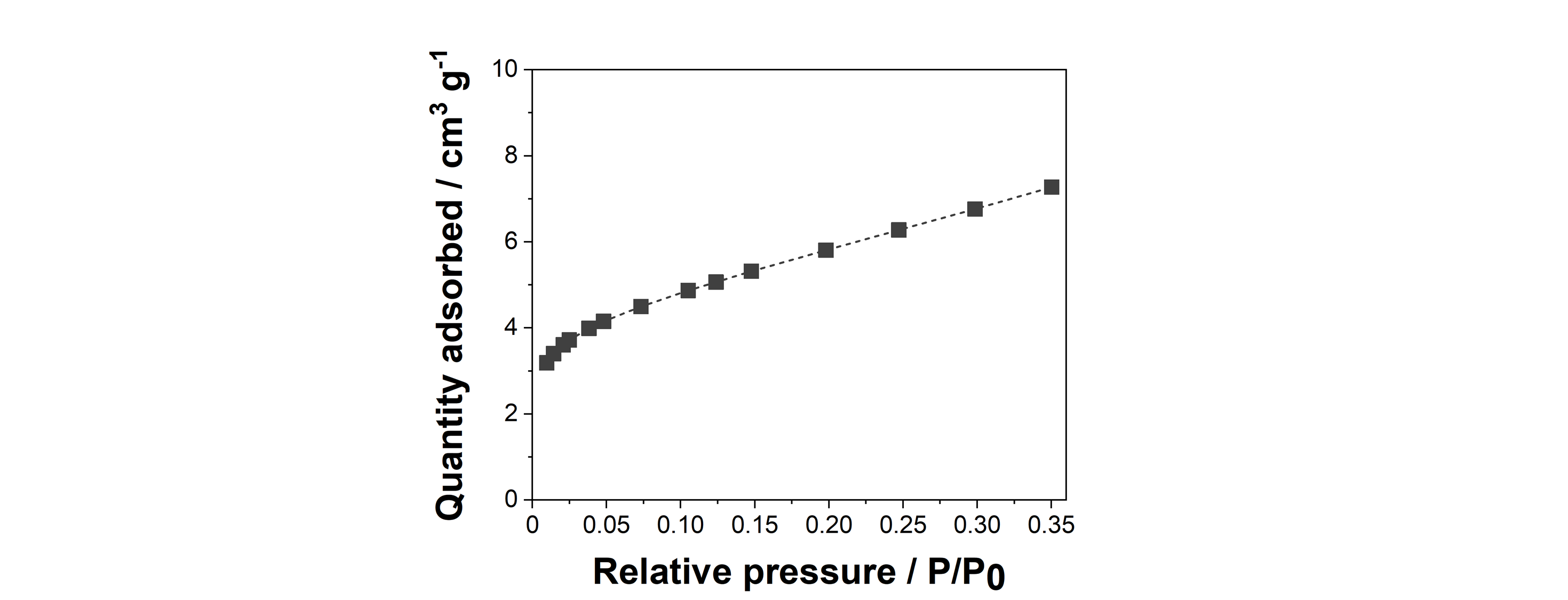


**Figure S3:** N_2_ adsorption-desorption isotherms and calculated BET surface areas of the as-received Raney Ni microparticles.


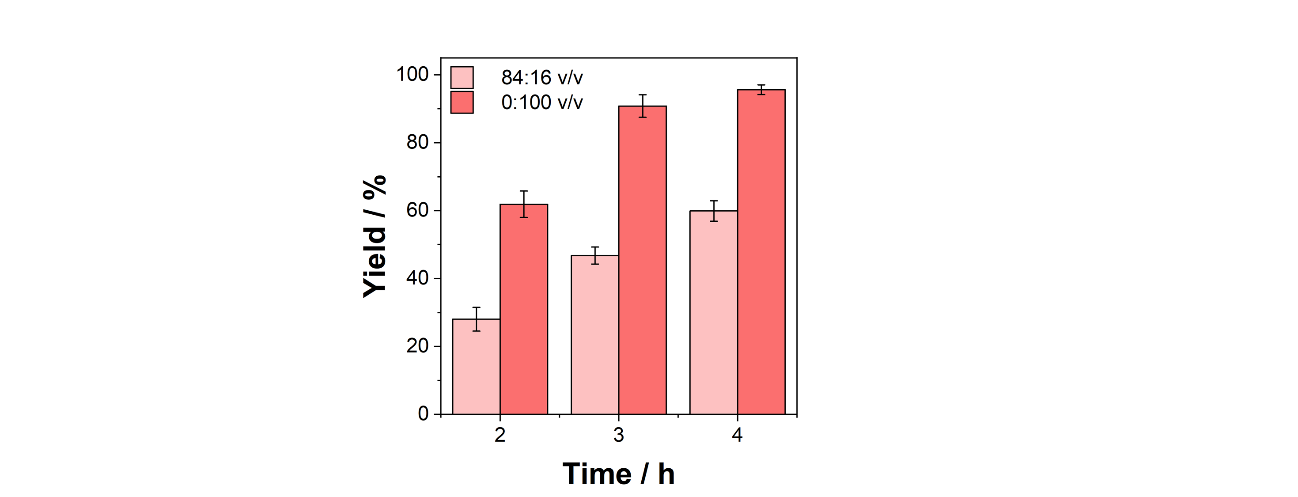


**Figure S4:** Effect of isopropanol content as the H-source in the yield of γ-valerolactone at different reaction times. Conditions: catalyst = 120 mg; [LA]_0_ = 0.5 M; T = 160 ºC. Each condition was performed in triplicate. Ratio: water:isopropanol v/v.


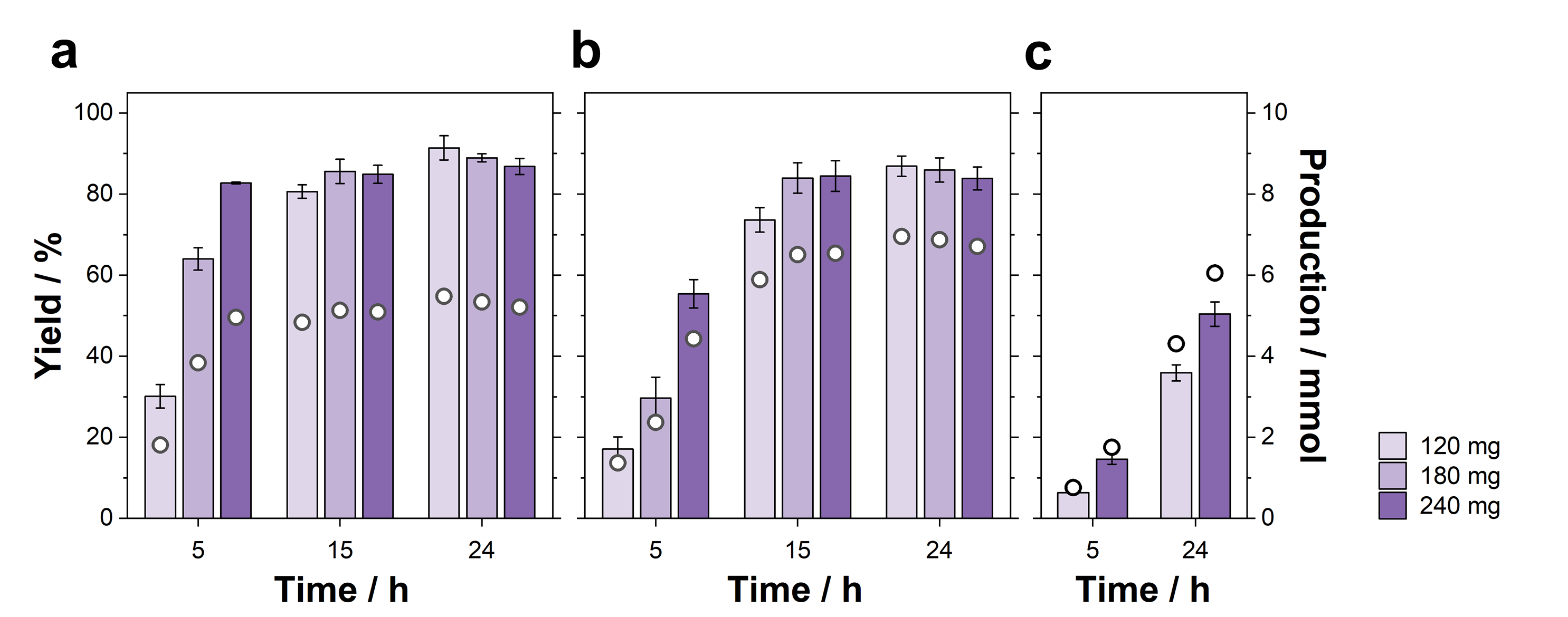


**Figure S5:** Evaluation of the yield (bars) and production (dots) of γ-valerolactone provided under solvothermal conditions (T = 160 ºC) at different reaction times and amount of catalyst at an initial LA concentration of: (a) 1.5 M, (b) 2 M and (c) 3 M. Each condition was performed in triplicate.


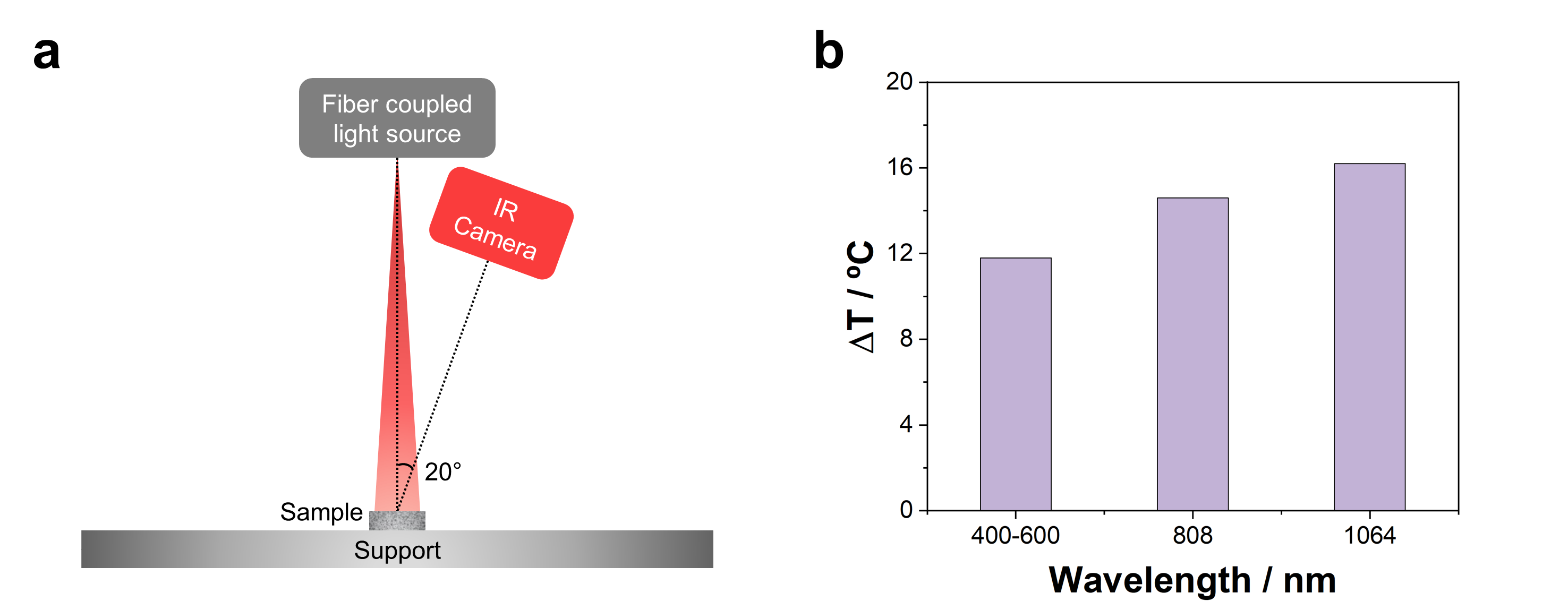
 **Figure S6:** a) Schematic of the photothermal set-up to characterize the photothermal response of the Raney Ni. b) Results of the photothermal analysis of dry Raney Ni powder (100 mg homogeneously distributed in an area of 0.64 cm^2^), illuminated by light intensity of 100 mW/cm^2^ of different wavelengths: white LED (400-600), and laser diodes with emission at 808 nm and 1064 nm.

**
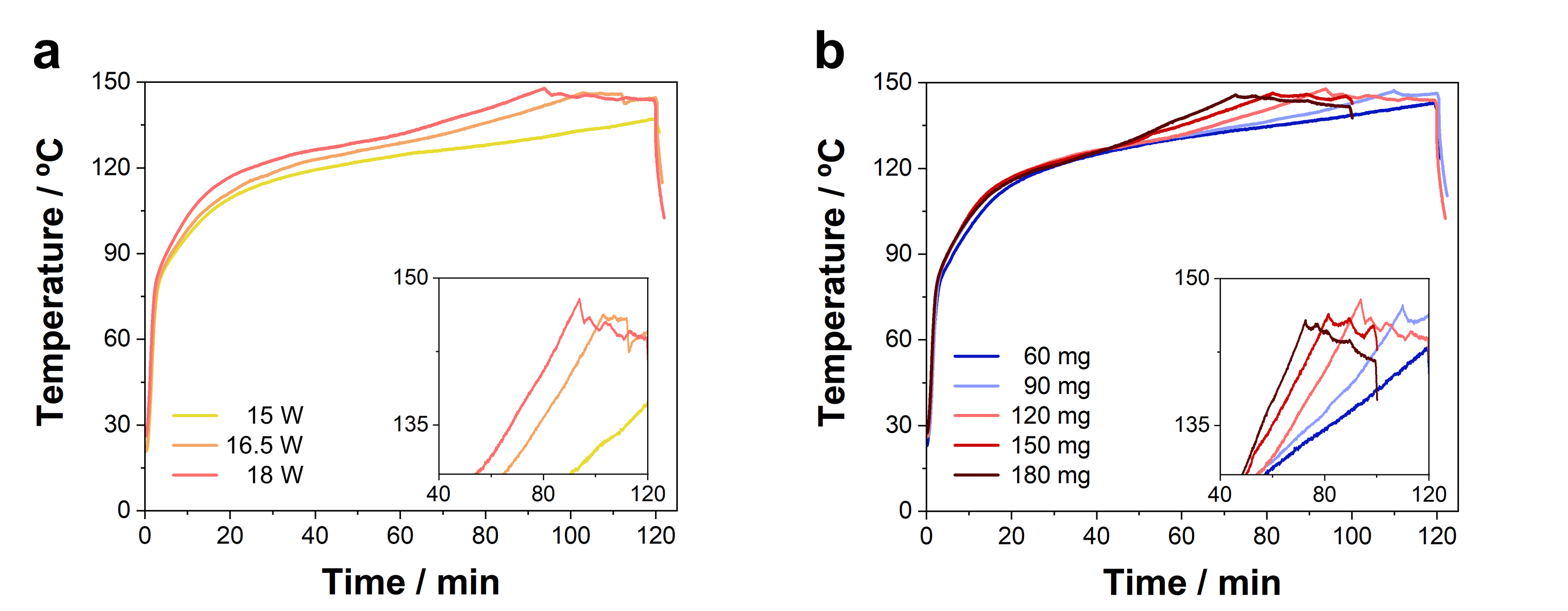
**

**Figure S7:** Dependence of the heating rate of the reaction medium ([LA]_0_ = 0.5 M) with respect to: (a) the laser power (catalyst = 120 mg) and (b) the amount of catalyst added to the reaction (laser power: 18 W).

**
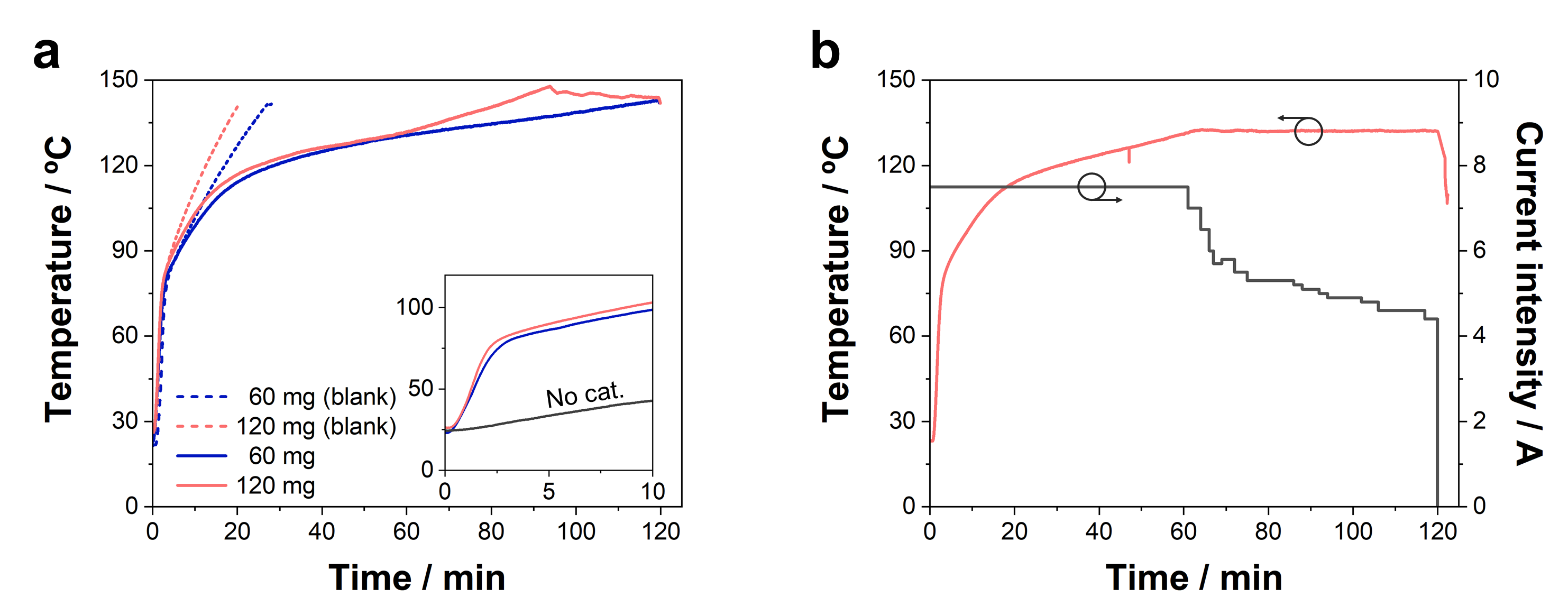
**

**Figure S8:** (a) Comparison of the heating rate between pure isopropanol (blank, dashed lines) and the reaction medium ([LA]_0_ = 0.5 M in isopropanol, solid lines) in the presence of different dosages of catalyst at a fixed laser power of 18 W (the inset shows the heating profile of the reaction medium without catalyst). (b) Temperature profile of a reaction conducted at a fixed maximum temperature of 132 °C and the laser power applied throughout the experiment (catalyst = 120 mg, laser initial power: 18 W).

**
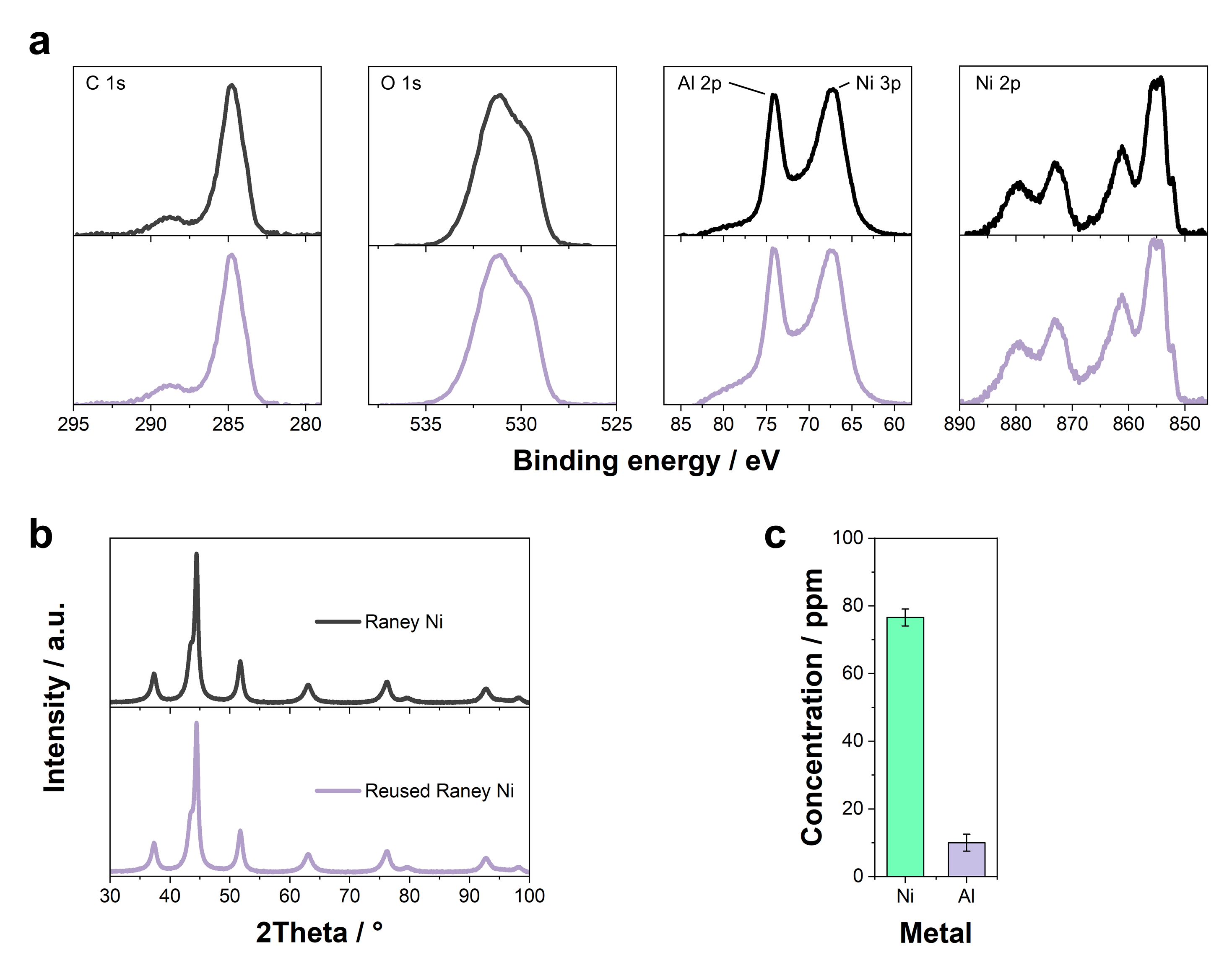
 Figure S9:** Effect of reusability over the catalyst after 5 cycles: (a) XPS and (b) XRD comparing the as-received Raney Ni microparticles (above) with the reused ones (below). (c) Concentration of metal ions detected within the reaction medium accumulated throughout 5 cycles.

**
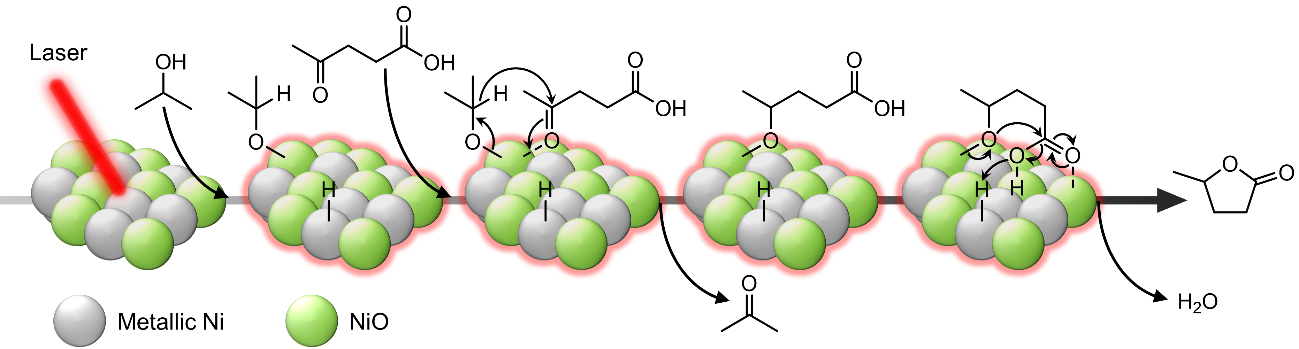
 Scheme S1:** Feasible mechanism for the hydrogenation and cyclization of levulinic acid to γ-valerolactone with isopropanol under photo-thermo-catalytic conditions over Raney Ni, based on the Meerwein−Ponndorf−Verley reaction. As proposed, metallic Ni acts as the active site for hydrogen while NiO serves as acid site to anchor carbonyl groups, facilitating hydrogen interchange and lactonization to γ-valerolactone from 4-hydroxypentanoate.
